# Nucleotide binding database NBDB – a collection of sequence motifs with specific protein-ligand interactions

**DOI:** 10.1093/nar/gkv1124

**Published:** 2015-10-26

**Authors:** Zejun Zheng, Alexander Goncearenco, Igor N. Berezovsky

**Affiliations:** 1 Bioinformatics Institute, Agency for Science, Technology and Research (A*STAR), 30 Biopolis Street, #07–01, Matrix, 138671, Singapore; 2 Computational Biology Unit, Department of Informatics, University of Bergen, Norway; 3 Department of Biological Sciences (DBS), National University of Singapore (NUS), 8 Medical Drive, 117579, Singapore

## Abstract

NBDB database describes protein motifs, elementary functional loops (EFLs) that are involved in binding of nucleotide-containing ligands and other biologically relevant cofactors/coenzymes, including ATP, AMP, ATP, GMP, GDP, GTP, CTP, PAP, PPS, FMN, FAD(H), NAD(H), NADP, cAMP, cGMP, c-di-AMP and c-di-GMP, ThPP, THD, F-420, ACO, CoA, PLP and SAM. The database is freely available online at http://nbdb.bii.a-star.edu.sg. In total, NBDB contains data on 249 motifs that work in interactions with 24 ligands. Sequence profiles of EFL motifs were derived *de novo* from nonredundant Uniprot proteome sequences. Conserved amino acid residues in the profiles interact specifically with distinct chemical parts of nucleotide-containing ligands, such as nitrogenous bases, phosphate groups, ribose, nicotinamide, and flavin moieties. Each EFL profile in the database is characterized by a pattern of corresponding ligand–protein interactions found in crystallized ligand–protein complexes. NBDB database helps to explore the determinants of nucleotide and cofactor binding in different protein folds and families. NBDB can also detect fragments that match to profiles of particular EFLs in the protein sequence provided by user. Comprehensive information on sequence, structures, and interactions of EFLs with ligands provides a foundation for experimental and computational efforts on design of required protein functions.

## INTRODUCTION

Nucleotide-containing ligands are indispensable in various biochemical transformations taking place in living cells (1). The ligands are comprised of several common chemical parts: nitrogenous bases, phosphate groups, ribose sugar and other moieties such as flavin and nicotinaminde. The chemical structure of ligands, particularly the presence of phosphate groups is responsible for their universal biological functions. For instance, ATP is well known for providing energy to enzymatic reactions, DNA repair machinery, cell division and activation of motor proteins (2). Transfer of phosphate groups in protein phosphorylation is a key mechanism in cell signaling, and many of these ligands are co-enzymes and essential vitamins (3,4). Importance of nucleotide-containing ligands in bioenergetics is reflected in high conservation of protein–ligand interactions, some of them resembling primordial nucleotide–peptide interactions in the origin of life (2). Walker A motif (or P-loop) responsible for interactions with the phosphate in nucleotides was among the first to be detected as a signature of nucleotide binding (5) and shown to be highly conserved (5–7). Sequence/structure determinants (8–10) of nucleotide binding along with evolutionary implications (11) have been considered for individual ligands (12–15) or small groups of them (16–18). Despite the great importance and in-depth studies of protein-DNA complexes reviewed elsewhere (19), comprehensive study of major nucleotide-containing ligands with description of their generic characteristics is still lacking. Crucial role of nucleotide-containing ligands in the diversity of cellular functions and rich evolutionary history of enzymes call for accurate and systematic study of these ligands and their interactions with different proteins. Based on the previous theoretical studies, it was hypothesized that all natural enzymes could be represented as combinations of elementary functional loops (EFLs)—presumable basic units of protein function (20,21). Therefore, it is of special interest to explore EFLs that specifically recognize ligands or interact with particular chemical groups, and to characterize their conserved contacts, functional signatures and sequence/structure determinants.

We present here a database of 249 profiles of EFLs that are involved in binding of different parts of the 24 most common nucleotide-containing ligands, including mononucleotides formed by nitrogenous bases adenosine, guanosine and cytosine: ATP, AMP, ATP, GMP, GDP, GTP, CTP and their derivatives PAP, PPS; flavin- and nicotinamide-containing nucleotides and dinucleotides: FMN, FAD(H), NAD(H), NADP. Additionally, the database includes cyclic nucleotides and dinucleotides such as cAMP, cGMP, c-di-AMP and c-di-GMP (22,23). For completeness we also include nucleotide-like molecules and coenzymes, such as ThPP, THD, F-420, ACO, CoA, PLP and SAM (for complete information on ligands see Supplementary Table S1 and ‘Help’ section on the NBDB website). The set of profiles of EFLs contains several very conserved archetypal signatures, for instance the generalized signatures GxxGxG and GxGxxG of phosphate binding in various nucleotides and dinucleotides, respectively. These patterns of glycines expose protein backbone in a particular way so that the main chain amide groups form hydrogen bonds with oxygens in ligand's phosphate groups. Other EFLs may interact with several chemical groups simultaneously, thereby facilitating molecular recognition and binding of specific ligands. We present here an example of a protein interacting with CTP ligand, illustrating how combinations of EFLs can form interactions with different parts of ligands.

The website http://nbdb.bii.a-star.edu.sg/ is a suite of interactive tools for exploratory analysis of ligands, profiles of EFLs and their interactions. Additionally, the search function allows one to identify ligand-binding sites in a given protein sequence. The NBDB database presented here can be used as a starting point in exploring different aspects of evolution of protein–nucleotide interactions, it can be of help in functional annotation of sequences and it can give valuable insights for designing the enzymes that bind specific ligands.

## THEORETICAL BACKGROUND AND COMPUTATIONAL METHODS

Based on our previous theoretical studies we proposed that all natural enzymes could be represented as combinations of EFLs—presumable basic units of protein function (20,21). Elementary function (EF) is defined as a chemical reaction step in enzymatically catalyzed biochemical transformation or binding of a substrate, product or intermediate molecule (24). Structural carriers of elementary functions—EFLs are defined as closed loops (returns of the polypeptide chain (25,26)), possessing one or few functional residues and bringing them to protein's active sites (20,21). Rigorous statistics of the PDB database prompted us to hypothesized that polymer nature of polypeptide chains determines common structural characteristics of EFLs: (i) shape – returns of the backbone; (ii) typical size of 25–30 amino acid residues (25,26). Further, we found strong indications that the most common EFLs with very basic and omnipresent elementary functions are apparently descendants of prebiotic ring-like peptides, which gave rise to the first enzymatic domains in the origin of life (2). Representatives of these EFLs and their evolutionary prototypes (20,21) can be found in different protein families, superfamilies and folds (27), allowing one to unravel deep evolutionary connections in the modern-day protein universe (2). These connections showed that the evolution of protein function (28,29) is complex, and, in addition to domain recombination, may have been driven by recombination of functional segments between protein domains (2,27,30,31). However, the specificity of molecular interactions in evolutionary conserved prototypes (20) and omnipresence of their descendants in protein folds and functions motivated us to develop a computational procedure for their derivation that differs from ancestral reconstruction due to the absence of any phylogenetic assumptions (20,21). In general, this procedure can derive profiles of elementary functions on different levels of conservation, from the most generic evolutionary prototypes (20) found in distant functional superfamilies and even in different protein folds to specific profiles of elementary functions in particular protein families (2,27). In brief, the procedure can be described as an iterative *de novo* derivation of sequence profiles in the form of position-specific scoring matrices from a collection of nonredundant sequences from UniProt, followed by their hierarchical clustering. The procedure's unique scoring function weights profile positions according to their information content, thus emphasizing on the importance of the functional signature. Due to the limited size of the profiles, the estimates of statistical significance of profile-sequence matches are based on the empirical distribution of scores obtained for the profile with shuffled positions. Here, we report one-sided p-values calculated for the z-scores of profile-sequence matches. Complete description of the procedure is available elsewhere (21), some relevant details are also provided on the NBDB website.

## DESCRIPTION OF THE DATABASE

The database is designed to provide fast and efficient access to the collection of sequence profiles of EFLs that bind most-common nucleotides and nucleotide-containing ligands. In particular, the database describes atomistic details of all hydrogen bonds between proteins and their cognate ligands. Thus, each position of the EFL profile is annotated by its interactions with different parts of ligands: phosphate and sulfate groups; ribose and other sugars; adenine, guanine and cytosine nitrogenous bases; acetyl, flavin, nicotinamide, pyridoxal and thiamin moieties. The database allows exploring the profiles of EFLs by the interacting ligand and ligand part, e.g. ribose in ADP. Each protein–ligand interaction is annotated by representative structural matches collected in the PDB database, with interacting proteins classified according to SCOP (32). The database allows searching for known EFLs and ligand binding sites given a protein sequence of interest. Below is a description of different pages and options available in the database along with a brief note on the implementation and usage.

## MATERIALS AND METHODS

Sequence origins for derivation of profiles of EFLs were obtained in two-step procedure from the PDB structures crystalized with the ligands of interest. First, the hydrogen bonding interactions were determined with the precise geometric criteria with tolerances of 0.4 angstroms and 20.0 degrees using UCSF Chimera v1.10.1 (33). Second, structural motifs that interact with the corresponding parts of ligands were determined and their sequences were used as origins in the profile derivation procedure. By following a procedure described in detail elsewhere (2,20) the origins were iteratively compared to Uniref50 sequences from UniProt release 2014_08 (34) as the nonredundant source of proteomic sequences, until they converged to sequence profiles.

The database contains 249 profiles of EFLs interacting with different parts of the following 24 nucleotide-containing ligands: AMP, ADP, ATP, GMP, GDP, GTP, CTP, CoA, Acelyl-CoA, FMN, F-420, FAD(H), NAD(H), NADP; cyclic nucleotides and dinucleotides: cAMP, cGMP, c-di-AMP, c-di-GMP; other biologically-relevant cofactors: SAM, PPS, PAP, PLP, ThPP, THD. According to SCOP (32) classification, representatives of ligand-binding EFLs are found in 74 folds, 84 superfamilies and 195 families.

## STRUCTURE OF THE DATABASE AND WEBSITE NAVIGATION

**Figure 1.**
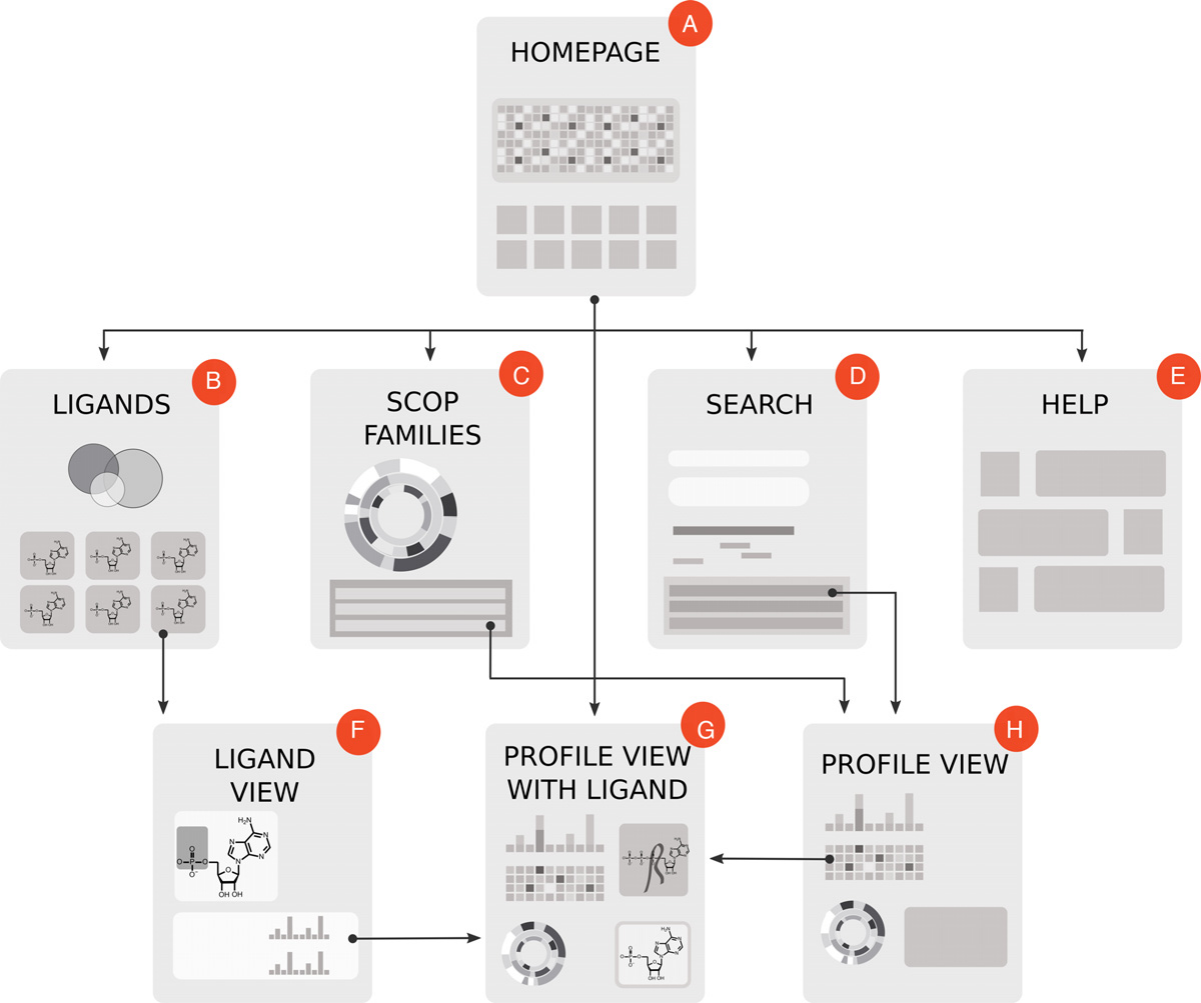
The sitemap scheme with logical connections between the elements of the database. There are eight main types of pages: (**A**) Homepage, (**B**) Ligands, (**C**) SCOP families, (**D**) Search, (**E**) Help, (**F**) Ligand view, (**G**) Profile view with ligand, (**H**) Profile view. Arrows show options for moving from one part of the data to another.

Figure 1 shows the database sitemap scheme with logical connections between the pages. There are eight main types of pages: (i) Homepage, (ii) Ligands page, (iii) SCOP families page, (iv) Search page, (v) Help page, (vi) Ligand view page, (vii) Profile with ligand view page, (viii) Profile view (without ligand) page. The main menu on top of each page provides links to (a–e).

***Homepage*** introduces user to the database and provides matrix navigation for an overview of all interactions in the database and quick access to the data. Its 24 rows designate the ligands and 249 columns correspond to the profiles of EFLs. Existing interactions between the profiles and ligands are marked by color. User can directly navigate to a profile's page with all relevant information describing its interaction with a particular ligand of interest.

***Ligands page*** shows the list of all ligands described in the database, their 2D structures and describes molecular moieties of each ligand. Clicking on the ligand leads to the ‘*Ligand view page*’ with detailed information about the selected molecule. We grouped ligands into several overlapping classes displayed on top of the page and allow filtering the list instantaneously according to the selected ligand class.

***Ligand view page*** shows the 2D view of the particular ligand's structure and allows user to select different chemical moieties of the ligand thereby focusing on interaction with a specific ligand's part. The table in the ligand view page shows the list of profiles of EFLs that bind to any (or selected by user) part of the ligand.

***SCOP families page*** contains an interactive circular diagram that shows a distribution of EFL profiles classified according to the SCOP hierarchy: class (inner circle), fold (second), superfamily (third) and family (outer circle). The segment sizes are proportional to the numbers of profiles in each SCOP class, fold, superfamily or family. Clicking on a segment within the circle shows a table with the corresponding set of EFL profiles.

***Search page*** requires a single protein sequence in FASTA format as an input. Optionally, user can provide a protein name or UniProt accession number, and the sequence will be automatically fetched. The search works similar to RPS-BLAST; it identifies all EFL profiles and visualizes a map of hits within the subject sequence. Each hit is also listed in the table. The profile-sequence search procedure is described elsewhere in detail (2). The p-value threshold is set to the recommended value of 10^−7^ in order to guarantee reliable output. Search takes less than a few seconds even for large sequences.

***Profile view page*** shows the EFL profile and all of its interactions. If the ligand is specified, it shows interactions with a particular ligand. The sequence profile is shown as a logo generated with the help of Weblogo software library (35). Profile name consists of the most conserved residues in its sequence signature. Profile can also be downloaded as a position frequency matrix. Below the profile, there is matrix of interactions with various ligands and their atoms aligned with corresponding positions of the profile. Colored cells in the matrix indicate interactions between protein motifs and atoms of the ligand. The color of a cell denotes different ligand moieties. Color intensity indicates the level of conservation of corresponding interactions. The ‘zoom in’ button below the ligand picture will show atom labels for the ligand. Circular diagram shows the distribution of families in SCOP for the EFL's profile. If profile is shown in the context of a ligand, it will be illustrated by the structural examples for a particular ligand listed in the table. Otherwise an illustration of the profile's structural representative is provided. In case the profile is displayed in the context of a ligand the table of structural examples includes the links to the PDB structure viewer. Additionally, PoseView (36) plots, which are downloaded directly from the PDB show 2D projections of all ligand–protein interactions in a given protein for the ligand of interest. Protein name input box accepts complex expressions with UniProt query syntax and allows a PDB identifier as an input, thus helping to identify all ligand-binding EFLs in a given structure.

## DATABASE USAGE

**Figure 2.**
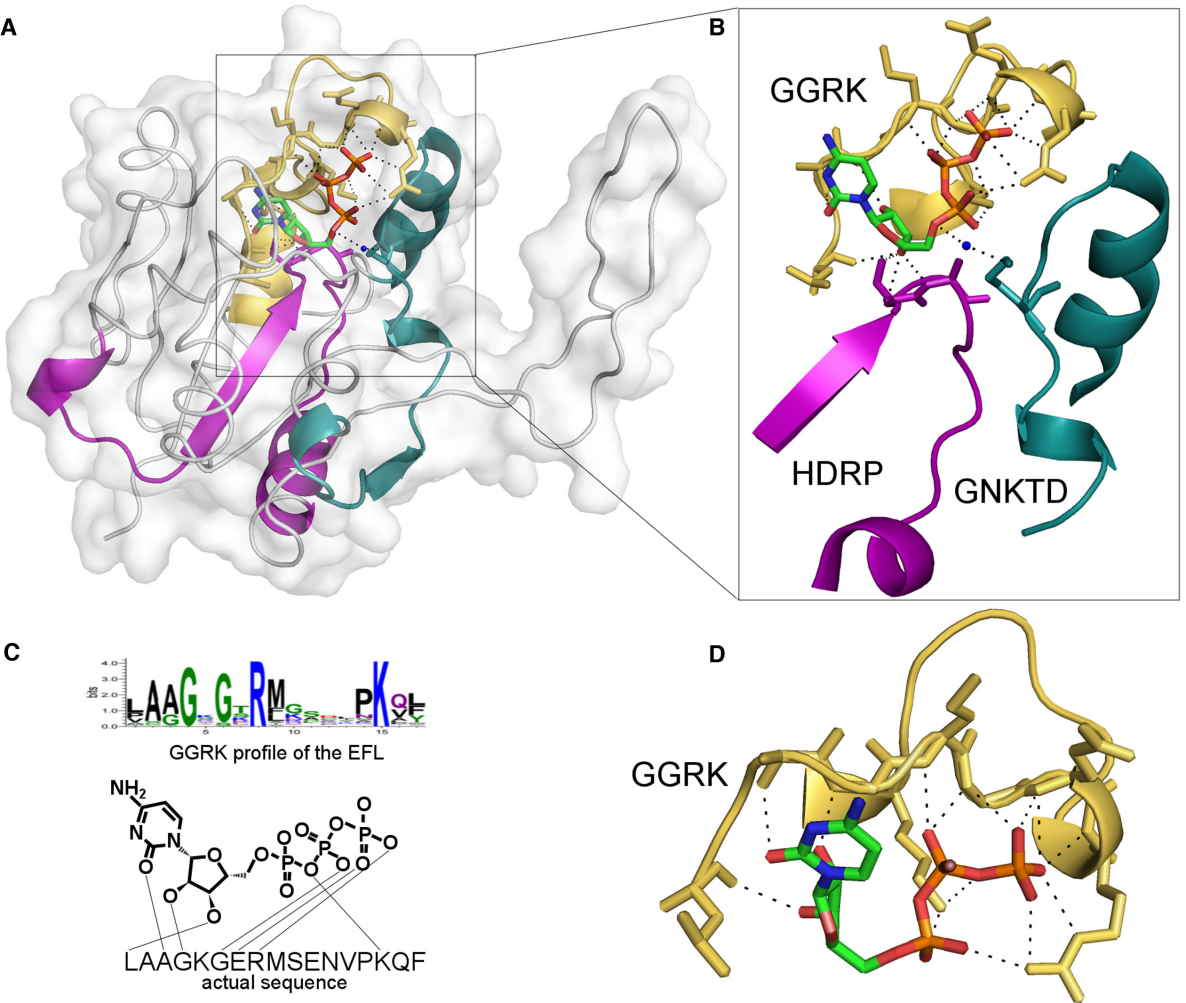
Structure of 2-C-methyl-D-erythritol 4-phosphate cytidylyltransferase from *Thermotoga maritima* with bound CTP. UniProt accession number for the protein is Q9 × 1B3, PDB ID 1vpa. (**A**) Structure with three ligand-binding EFLs are displayed as colored ribbons. (**B**) Zoom-in to the CTP binding site. (**C**) Scheme of contact between sequence found by the ‘GGRL’ profile. (**D**) Structure of the motif (yellow) found by the ‘GGRK’ profile. This motif interacts with three parts of the ligand: base, ribose and phosphate groups. Motif found by the ‘HDRP’ profile interacts with ribose (magenta). Motif found by ‘GNKTD’ profile (green loop) makes a contact with phosphate groups via the water molecule.

**Figure 3.**
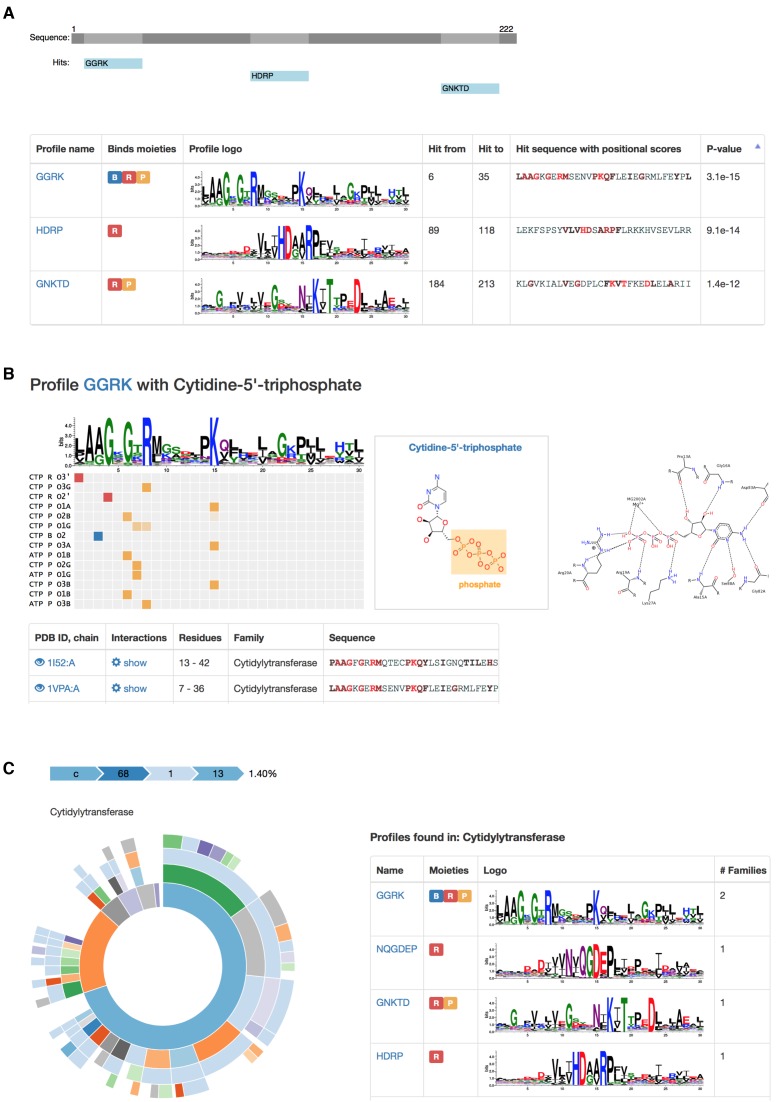
Screenshots with examples of data obtained for CTP ligand. **(A)** Sequence search results for cytidyltransferase; The list of profile hits and their sequence matches are provided in the table, and the hits are mapped onto the subject sequence. **(B)** EFL profile GGRK that binds phosphate in Cytidine-5′-triphosphate (CTP). The matrix shows all interacting ligands, ligand parts and atoms with CTP triphosphate group highlighted in orange; The table below shows structural examples of interactions with a 2D interaction plot generated by PoseView (right). **(C)** SCOP family view for Cytidyltransferase family shows the list of EFL profiles found in the family.

Altogether, the data collected in this database provides a detailed picture of important interactions (and their sequence/structure determinants) that work in binding of nucleotide-containing ligands and biologically relevant cofactors. Below we describe an example of 2-C-methyl-D-erythritol 4-phosphate cytidylyltransferase from *Thermotoga maritima* with bound Cytidine-5′-triphosphate (CTP). Figure 2 shows the protein structure (panel a) with three ligand-binding EFLs displayed as colored ribbons. Figure 2B is a zoom-in to the binding site with three motifs found by the profiles ‘GGRK’ (yellow), ‘HDRP’ (magenta) and ‘GNKTD’ (green) loops, respectively. Yellow loop interacts with three parts of the ligand: base, ribose and phosphate groups (Figure 2C and D). Magenta EFL (found by the ‘HDRP’ profile) interacts with ribose (magenta), and green EFL (‘GNKTD’ profile) makes contacts with phosphate groups via a water molecule. These three EFLs found in one protein illustrate a comprehensive character of the database, showing how diversity of the accumulated data can help user build a detailed picture of interactions between the ligand of interest and the target protein. Figure 3 contains screenshots, showing examples of different data and outputs that can be obtained for CTP (ligand in the protein discussed in Figure 2). It starts from results of the sequences search for cytidyltransferase (Figure 3A), where hits are mapped onto the query sequence, and the list of profile hits and their sequence matches are provided in the table. Figure 3B contains data on the profile GGRK that binds phosphate in Cytidine-5′-triphosphate (CTP). SCOP family view for Cytidyltransferase family shows the list of EFL profiles found in the family (Figure 3C).

## CONCLUSION

We believe that the most important advantage of this database stems from the strong theoretical foundation of the EFLs and completeness and the level of detail about interactions of representative EFLs with nucleotide-containing ligands. The most common EFLs were proposed to be most likely descendants of the ancient ring-like peptides, which served as basic building blocks of the first enzymatic domains. Modern proteins and their EFLs follow the basic rules and requirements established in the very beginning of the protein evolution, hence many enzymatic functions can be considered as combinations of the corresponding EFLs. The NBDB database provides a comprehensive set of 249 EFL profiles that interact with 24 nucleotide-containing ligands and other relevant cofactors. The set of profiles contains different entities, starting from the very common and ancient signatures existing from the origin of life (e.g. profiles of the phosphate binding in dinucleotides and nucleotides, GxGxxG and GxxGxG) and ending with distinct signatures that work in more specific ligands. Since interaction with nucleotide-containing ligand is a key element of many biochemical transformations and signaling processes, we expect that the database will be of great help for researcher working on different aspect of protein function, its evolution and design. Intended expansion of the EFLs collection and characterization of other elementary functions will provide an important theoretical background for experimental efforts in design of required protein functions.

## References

[1] Holliday G.L., Andreini C., Fischer J.D., Rahman S.A., Almonacid D.E., Williams S.T., Pearson W.R. (2012). MACiE: exploring the diversity of biochemical reactions. Nucleic Acids Res..

[2] Goncearenco A., Berezovsky I.N. (2015). Protein function from its emergence to diversity in contemporary proteins. Phys. Biol..

[3] Fischer J.D., Holliday G.L., Rahman S.A., Thornton J.M. (2010). The structures and physicochemical properties of organic cofactors in biocatalysis. J. Mol. Biol..

[4] Fischer J.D., Holliday G.L., Thornton J.M. (2010). The CoFactor database: organic cofactors in enzyme catalysis. Bioinformatics.

[5] Walker J.E., Saraste M., Runswick M.J., Gay N.J. (1982). Distantly related sequences in the alpha- and beta-subunits of ATP synthase, myosin, kinases and other ATP-requiring enzymes and a common nucleotide binding fold. EMBO J..

[6] Smith C.A., Rayment I. (1996). Active site comparisons highlight structural similarities between myosin and other P-loop proteins. Biophys. J..

[7] Kinoshita K., Sadanami K., Kidera A., Go N. (1999). Structural motif of phosphate-binding site common to various protein superfamilies: all-against-all structural comparison of protein-mononucleotide complexes. Protein Eng..

[8] Brakoulias A., Jackson R.M. (2004). Towards a structural classification of phosphate binding sites in protein-nucleotide complexes: an automated all-against-all structural comparison using geometric matching. Proteins.

[9] Gherardini P.F., Ausiello G., Russell R.B., Helmer-Citterich M. (2010). Modular architecture of nucleotide-binding pockets. Nucleic Acids Res..

[10] Stegemann B., Klebe G. (2012). Cofactor-binding sites in proteins of deviating sequence: comparative analysis and clustering in torsion angle, cavity, and fold space. Proteins.

[11] Xie L., Bourne P.E. (2008). Detecting evolutionary relationships across existing fold space, using sequence order-independent profile-profile alignments. Proc. Natl. Acad. Sci. U.S.A..

[12] Carugo O., Argos P. (1997). NADP-dependent enzymes. I: Conserved stereochemistry of cofactor binding. Proteins.

[13] Carugo O., Argos P. (1997). NADP-dependent enzymes. II: Evolution of the mono- and dinucleotide binding domains. Proteins.

[14] Denessiouk K.A., Johnson M.S. (2003). ‘Acceptor-donor-acceptor’ motifs recognize the Watson-Crick, Hoogsteen and Sugar ‘donor-acceptor-donor’ edges of adenine and adenosine-containing ligands. J. Mol. Biol..

[15] Dym O., Eisenberg D. (2001). Sequence-structure analysis of FAD-containing proteins. Protein Sci..

[16] Denessiouk K.A., Rantanen V.V., Johnson M.S. (2001). Adenine recognition: a motif present in ATP-, CoA-, NAD-, NADP-, and FAD-dependent proteins. Proteins.

[17] Kleiger G., Eisenberg D. (2002). GXXXG and GXXXA motifs stabilize FAD and NAD(P)-binding Rossmann folds through C(alpha)-H… O hydrogen bonds and van der waals interactions. J. Mol. Biol..

[18] Traut T.W. (1994). The functions and consensus motifs of nine types of peptide segments that form different types of nucleotide-binding sites. Euro. J. Biochem..

[19] Gromiha M.M., Nagarajan R. (2013). Computational approaches for predicting the binding sites and understanding the recognition mechanism of protein-DNA complexes. Adv. Protein Chem. Struct. Biol..

[20] Goncearenco A., Berezovsky I.N. (2010). Prototypes of elementary functional loops unravel evolutionary connections between protein functions. Bioinformatics.

[21] Goncearenco A., Berezovsky I.N. (2011). Computational reconstruction of primordial prototypes of elementary functional loops in modern proteins. Bioinformatics.

[22] Chou S.H., Galperin M.Y. (2015). Diversity of c-di-GMP-binding proteins and mechanisms. J. Bacteriol..

[23] Gomelsky M., Galperin M.Y. (2013). Bacterial second messengers, cGMP and c-di-GMP, in a quest for regulatory dominance. EMBO J..

[24] Jencks W.P. (1987). *Catalysis in Chemistry and Enzymology*.

[25] Berezovsky I.N., Grosberg A.Y., Trifonov E.N. (2000). Closed loops of nearly standard size: common basic element of protein structure. FEBS Lett..

[26] Berezovsky I.N., Trifonov E.N. (2001). Van der Waals locks: loop-n-lock structure of globular proteins. J. Mol. Biol..

[27] Goncearenco A., Berezovsky I.N. (2012). Exploring the evolution of protein function in Archaea. BMC Evol. Biol..

[28] Galperin M.Y., Koonin E.V. (2012). Divergence and convergence in enzyme evolution. J. Biol. Chem..

[29] Galperin M.Y., Makarova K.S., Wolf Y.I., Koonin E.V. (2015). Expanded microbial genome coverage and improved protein family annotation in the COG database. Nucleic Acids Res..

[30] Caetano-Anolles G., Sun F.J., Wang M., Yafremava L.S., Harish A., Kim H.S., Knudsen V., Caetano-Anolles D., Mittenthal J.E. (2008). Origins and evolution of modern biochemistry: insights from genomes and molecular structure. Front. Biosci..

[31] Caetano-Anolles G., Yafremava L.S., Gee H., Caetano-Anolles D., Kim H.S., Mittenthal J.E. (2009). The origin and evolution of modern metabolism. Int. J. Biochem. Cell Biol..

[32] Murzin A.G., Brenner S.E., Hubbard T., Chothia C. (1995). SCOP: a structural classification of proteins database for the investigation of sequences and structures. J. Mol. Biol..

[33] Mills J.E., Dean P.M. (1996). Three-dimensional hydrogen-bond geometry and probability information from a crystal survey. J. Compute Aided Mol. Des..

[34] Hunter S., Apweiler R., Attwood T.K., Bairoch A., Bateman A., Binns D., Bork P., Das U., Daugherty L., Duquenne L. (2009). InterPro: the integrative protein signature database. Nucleic Acids Res..

[35] Crooks G.E., Hon G., Chandonia J.M., Brenner S.E. (2004). WebLogo: a sequence logo generator. Genome Res..

[36] Stierand K., Rarey M. (2007). From modeling to medicinal chemistry: automatic generation of two-dimensional complex diagrams. ChemMedChem.

